# Effects of Alpine Natural Health Resources on Human Health and Wellbeing

**DOI:** 10.3390/ijerph20126144

**Published:** 2023-06-16

**Authors:** Arnulf Josef Hartl, Johanna Freidl, Daniela Huber

**Affiliations:** Institute of Ecomedicine, Paracelsus Medical University Salzburg, Strubergasse 21, 5020 Salzburg, Austria; johanna.freidl@pmu.ac.at (J.F.); daniela.huber@pmu.ac.at (D.H.)

As humanity becomes progressively urban, a huge number of people could lose the opportunity to benefit from or develop an appreciation for nature [1,2]. This physical disconnection from natural environments has a diametric impact on health and wellbeing: Urban areas are associated with a number of health risk factors, such as air and noise pollution or overcrowding [3]. In addition, increasing urbanization is accompanied by a potential loss of contact with nature and biodiversity [1], and changes in lifestyle habits such as physical inactivity [4,5], increased stress factors [6] and changes in nutrition [7] and leisure behavior [4]. The consequences are rapid and the risk for noncommunicable chronic diseases and mental illness or distress is rising—health science studies increasingly show significant connections between differences in environment and poor physiological and psychological health [3,8,9]. Furthermore, data from the European Union show that rural dwellers under constant socioeconomic factors present a considerably higher level of life satisfaction compared with the urban population [10]. In this way, urban society demonstrates a continuously growing need for recreation and a reconnection with nature [11]. The COVID-19 pandemic even highlighted the importance of contact with nature to maintain human health [12].

The Alpine region, with its unique nature, terrain, climate, and cultural heritage, offers numerous opportunities to promote human health and wellbeing [13,14,15,16,17]. Regarding the growing scientific evidence on the health effects of natural resources and exercise in natural environments (“green exercise”) [18], there is a huge potential for the development of evidence-based recreation and therapies, as well as tourism products and service chains in the Alpine region that respond to specific health tourist demands [19].

This Special Issue includes relevant, high-quality papers highlighting actual and mainly novel factors concerned with “Effects of Alpine Natural Health Resources on Human Health and Wellbeing”. Nine original papers were published in this Special Issue, with one of them being a study protocol.

Schmude et al. [20] examined the success factors of health tourism based on natural attractions in selected European spa and health destinations. Here, natural resources such as water, salt, and air were assigned a central role in this context, as their evidence-based effects are highly relevant for the health and wellbeing of tourists.

Haid et al. [21] analyzed motives for cycling in the Alpine region and focused on the relative importance of health promotion and the influence of person-specific characteristics on it. Opportunities to advertise cycling tourism were derived from these motifs and characteristics.

In their research, Niedermeier et al. [22] focused on the question of a potential positive effect of alpine sport on development in adolescence. They discussed whether the characteristics of alpine sports have a trigger for a higher experience of agency, consequently satisfying the increased need for autonomy and independence in this age group.

Another important aspect of exercise in alpine surroundings was examined by Niebauer et al. [23], namely, the leading cause of non-traumatic deaths during downhill skiing and mountain hiking: the risk of sudden cardiac death. Differences in the risk factor patterns of this pathology were presented, and requirements for the physical fitness of skiers and hikers were discussed, which can support medical preventive advice.

In their study, Huber et al. [24] examined whether a hiking program with or without mental coaching has an impact on the cardiorespiratory parameters and quality of life of couples with sedentary behavior.

Likewise, Pichler et al. [25] defined people with a sedentary and inactive lifestyle as a group of interest: In their study protocol, they describe how they surveyed the influence of two types of nature-based therapy (nature connection therapy versus moderate mountain hiking) on physiological, psychological, and immunological parameters. Based on this, the long-term results of this study are presented: Forest therapy and mountain hiking could be safe and health-promoting interventions for high-functioning individuals with sedentary lifestyles, with women in particular benefiting even more [26].

Eisenberger et al.’s [27] findings are also interesting: The authors found that the criterion validity of Borg’s rating of perceived exertion in mountain hiking depends on the natural environment. Therefore, objective parameters for training control, such as heart rate, should be added to this type of intervention to improve intensity prescription and health safety.

Toussaint et al. [28] investigated the relationship between patient expectations and health-related quality of life in patients with multiple diseases and chronic pain who sought treatment at the Gasteiner Heilstollen in Böckstein near Bad Gastein, Austria.

We were invited as guest editors by the renowned *International Journal of Environmental Research and Public Health* to accompany the review and publication process of these articles. Therefore, we would like to take this opportunity to thank the Editorial Board and the Journal Office for their valuable work. We would also like to express our sincere thanks to all the authors, co-authors, and referees for their high-quality contributions.
